# Determinants of Implementation of a Critical Care Registry in Asia: Lessons From a Qualitative Study

**DOI:** 10.2196/41028

**Published:** 2023-03-06

**Authors:** Timo Tolppa, Vrindha Pari, Christopher Pell, Diptesh Aryal, Madiha Hashmi, Maryam Shamal Ghalib, Issrah Jawad, Swagata Tripathy, Bharath Kumar Tirupakuzhi Vijayaraghavan, Abi Beane, Arjen M Dondorp, Rashan Haniffa, Collaboration of Research Implementation & Training in Critical Care in Asia Investigators

**Affiliations:** 1 Network for Improving Critical Care Systems and Training Colombo Sri Lanka; 2 Chennai Critical Care Consultants Group Chennai India; 3 Amsterdam Institute for Global Health and Development Amsterdam Netherlands; 4 Department of Global Health Amsterdam UMC location University of Amsterdam Amsterdam Netherlands; 5 Hospital for Advanced Medicine and Surgery Kathmandu Nepal; 6 Ziauddin University Karachi Pakistan; 7 Wazir Akbar Khan Hospital Kabul Afghanistan; 8 Department of Anaesthesia and Intensive Care Medicine All India Institute of Medical Sciences Bhubaneswar India; 9 Critical Care Medicine Department Apollo Hospital Chennai India; 10 Indian Registry of IntenSive Care Chennai India; 11 Mahidol Oxford Tropical Medicine Research Unit Bangkok Thailand; 12 Nuffield Department of Medicine Oxford University Oxford United Kingdom; 13 See Acknowledgments

**Keywords:** CCU, critical care, registry, implementation, qualitative research, stakeholders, South Asia, health care

## Abstract

**Background:**

The Collaboration for Research, Implementation, and Training in Critical Care in Asia (CCA) is implementing a critical care registry to capture real-time data to facilitate service evaluation, quality improvement, and clinical studies.

**Objective:**

The purpose of this study is to examine stakeholder perspectives on the determinants of implementation of the registry by examining the processes of diffusion, dissemination, and sustainability.

**Methods:**

This study is a qualitative phenomenological inquiry using semistructured interviews with stakeholders involved in registry design, implementation, and use in 4 South Asian countries. The conceptual model of diffusion, dissemination, and sustainability of innovations in health service delivery guided interviews and analysis. Interviews were coded using the Rapid Identification of Themes from Audio recordings procedure and were analyzed based on the constant comparison approach.

**Results:**

A total of 32 stakeholders were interviewed. Analysis of stakeholder accounts identified 3 key themes: innovation-system fit; influence of champions; and access to resources and expertise. Determinants of implementation included data sharing, research experience, system resilience, communication and networks, and relative advantage and adaptability.

**Conclusions:**

The implementation of the registry has been possible due to efforts to increase the innovation-system fit, influence of motivated champions, and the support offered by access to resources and expertise. The reliance on individuals and the priorities of other health care actors pose a risk to sustainability.

## Introduction

The Lancet Global Health Commission on High-Quality Health Systems concluded that high-quality health systems could save over 8 million lives in low- and middle-income countries (LMICs) each year [1]. The ability to capture and use data to drive research and practice improvement is a core element of any high-quality health system [2]. Robust real-time information systems and registries, as well as individuals with strong research skills, underpin the effective use of data for learning through service evaluation, quality improvement, and clinical studies [1,2]. Unfortunately, the infrastructure to capture real-time data is largely absent from health systems in LMICs, and this remains a substantial barrier to the improvement of care [1,3].

The Wellcome-funded Collaboration for Research, Implementation, and Training in Critical Care in Asia (CCA) is a community of practice seeking to address this absence by implementing a cloud-based critical care registry distributed through nationally owned networks in 9 Asian countries [4]. The registry has been co-designed with stakeholders using an agile approach, whereby development is driven by user feedback [5-7]. A core set of data is captured contemporaneously with clinical care, enabling real-time feedback [8]. Characteristic of modern, high-quality registries, the CCA registry is responsive to evolving priorities and has facilitated observational research, pandemic surveillance, and clinical trials [9-11].

Implementing registries and health information systems to support high-quality health systems is complex, and varied outcomes have been reported [12,13]. Researchers have examined determinants of registry implementation, focusing largely on experiences in high-income settings and on small-scale projects in LMICs driven by stakeholders from high-income countries [14-18]. In LMICs, challenges to implementation have included limited local buy-in, a lack of technical expertise, inadequate hardware, and an unstable power supply [3,16,17]. Little research has examined how organizational cultures, health system priorities, and infrastructure influence registry implementation in LMICs, and thus further exploration is warranted [14,19,20]. There are also recent calls, including from the Lancet Commission on High-Quality Health Systems and recent expert commentaries, to study data use within health systems in diverse settings and examine the factors influencing the scale-up of digital health innovation in practice [1,2,18,21-23].

Drawing on interviews with stakeholders involved in the design, implementation, and use of the CCA registry, this article examines the determinants of implementation by examining processes of diffusion, dissemination, and sustainability of the registry in 4 South Asian countries.

## Methods

### Study Design

The study team conducted a qualitative phenomenological inquiry using semistructured interviews, drawing on the conceptual model of diffusion, dissemination, and sustainability of innovations in health service delivery [14]. The conceptual model comprises 7 key domains that determine successful diffusion, dissemination, and sustainability of innovations: innovation attributes, adopters and adoption process, communication and influence, inner context, outer context, implementation and sustainability, and linkage between components of the model [14]. This model was selected for its holistic, real-world approach.

### Innovation

The innovation is a critical care registry, a cloud-based mobile and desktop data capture application used to provide real-time data on care activity, case mix, and outcomes. Data are collected by designated data collectors (DCs), who are either clinical or nonclinical site staff or affiliated with the national registry team. The registry is independent of hospital information systems and, as such, represents an additional tool for sites to use for their own priorities, including service evaluation and research. The registry is described further in Multimedia Appendix 1 using the Template for Intervention Description and Replication [24].

### Settings

The study was conducted in 4 countries within the CCA that expressed interest in participating in the research: India, Nepal, Pakistan (lower-middle–income economies), and Afghanistan (low-income economy). Hospital beds per capita are estimated at fewer than 200 per 100,000 for all 4 countries [25]. There are 1.5, 2.3, and 2.8 adult critical care beds per 100,000 population in Pakistan, India, and Nepal, respectively [25]. Data on critical care beds are not currently available for Afghanistan.

Each country in the network adapts the CCA registry to their priorities and manages it as their own national registry with financial support from the CCA. The Pakistan Registry of Intensive CarE (PRICE) commenced in August 2017 and now includes 70 units [6]. The Indian Registry of IntenSive Care (IRIS) was established in January 2019 and has 34 contributing units [7]. The Nepal Intensive Care Registry Foundation (NICRF) started in September 2019 and currently includes 14 units. The critical care registry was initiated in Afghanistan in August 2020 and, at present, covers 20 units. The characteristics of the included sites and registries are outlined in Multimedia Appendix 1.

### Participants

**Figure 1:**
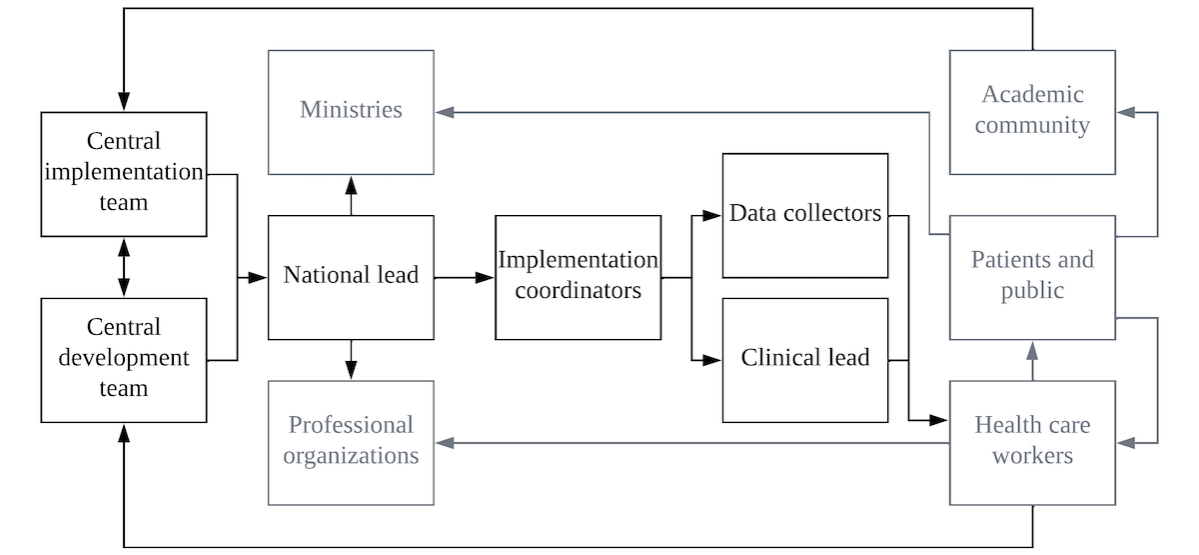
Organogram of the Collaboration for Research, Implementation, and Training in Critical Care in Asia team structure and stakeholders. The stakeholder groups invited to participate in the study are detailed in black.

The stakeholders invited to participate in the study represented the entire registry development team (DT) and implementation team (IT), as well as clinical leads (CLs) and DCs from a convenience sample of participating units (Figure 1). Data saturation was achieved with the planned convenience sample, so no further individuals were interviewed. Stakeholder roles and the implementation process are described in Multimedia Appendix 1.

### Ethics Approval

The study was approved by the Oxford Tropical Research Ethics Committee (reference 544-20). Participants were invited to participate via email, informed of the study objectives and voluntary nature of participation, and provided audio-recorded informed verbal consent. Participant confidentiality was ensured by not including identifying information in written notes and storing all study materials on password-protected electronic files. This study is reported in accordance with the Standards for Reporting Qualitative Research (Multimedia Appendix 1) [26].

### Procedures

Interviews were conducted between June 2020 and February 2021 via web-based audio conferencing (Zoom Video Communications Inc) by 3 trained researchers (IJ, TT, and VP) in English or by a coinvestigator in the local language using a translated interview guide. IJ and TT have clinical experience in critical care and were involved in the CCA registry’s implementation and design. VP has experience conducting qualitative research in Asia but was not involved in registry implementation. The interview guide (Multimedia Appendix 1) was informed by the domains of the conceptual model adapted to the characteristics of the registry [14]. Prompts were added throughout the study to explore emerging themes.

All interviews were audio-recorded and coded with the Rapid Identification of Themes from Audio recordings procedure [27]. Recordings were divided into 3-minute segments, each of which was coded using domains of the conceptual model, and deidentified notes were directly entered into the extraction table. Non-English interviews were reviewed in the local language and coded by the original interviewer together with VP, who entered notes into the extraction table in English [28].

### Data Analysis

Coded data were analyzed based on the constant comparative approach [29]. Comparisons were drawn across different sites and stakeholders, and attention was paid to conflicting accounts and outliers. Trustworthiness was enhanced by the involvement of additional team members in the analysis and refinement of themes, including those with extensive experience in qualitative research (CP and AB) and those leading the CCA registry development (RH). Debriefs between interviews and the inclusion of researchers without involvement in registry implementation were used to challenge existing assumptions. Credibility was enriched by checks to ensure themes were adequately comprehensive and by respondent checking at the end of each interview.

## Results

### Overview

A total of 32 participants were interviewed (Table 1): 15 site-level staff (CLs and DCs); 8 national IT members (national leads [NLs] and implementation coordinators); and 9 members of the central registry IT and DT. All the individuals who were approached agreed to participate. More male participants were interviewed (62.5%), and participants' time working with the CCA registry ranged from 2 weeks to over 2 years. Interviews lasted between 32 and 84 (mean 51.5 min, SD 11.8) minutes. In total, 27.5 hours of interviews were analyzed.

**Figure 2:**
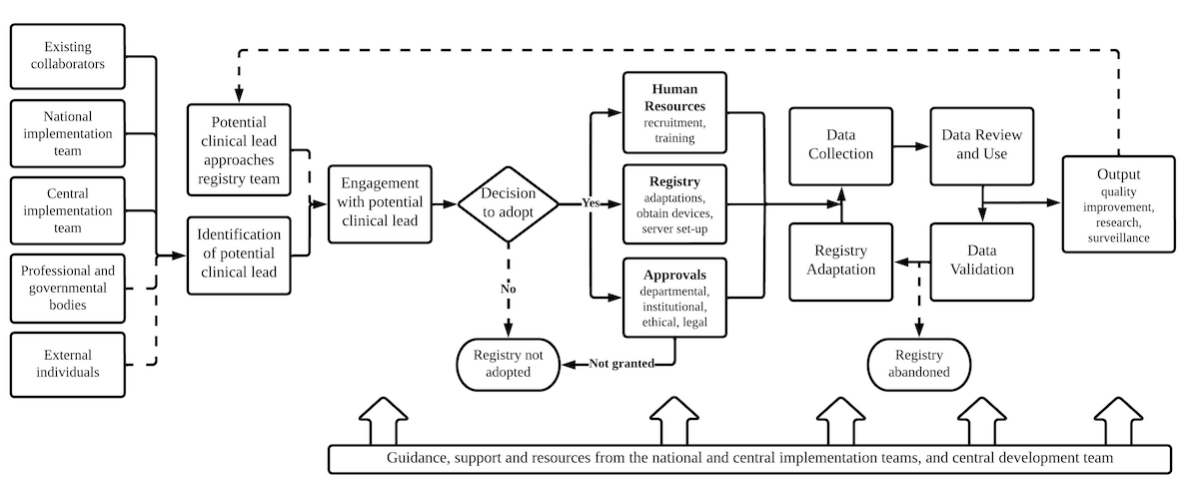
Implementation of the Collaboration for Research, Implementation, and Training in Critical Care in Asia registry as reported by participants (dashed lines represent less frequent processes).

Figure 2 summarizes the implementation of the registry as characterized by participants. Registries in India, Nepal, and Pakistan were started by NLs by approaching the registry team, whereas the NL in Afghanistan was identified through existing collaborations. In-country diffusion and dissemination occurred through NLs identifying potential CLs via existing collaborators, or less frequently, clinicians would approach the registry team after seeing output, such as a publication. Once a CL decided to adopt the registry, approvals were sought and resources were put in place prior to commencing data collection. All NLs and IT members provided examples of how registry adoption was either delayed or blocked due to difficulties in gaining approvals from hospital management. Once collected, clinical data were reviewed for quality and completeness using dashboards, and some CLs used the data for management reports and research output. Data validation and registry adaptation to site requirements (eg, unit of measure changes) occurred daily at the start of its use and subsequently weekly or monthly. Participants described 1 instance of a unit abandoning the registry.

**Figure 3:**
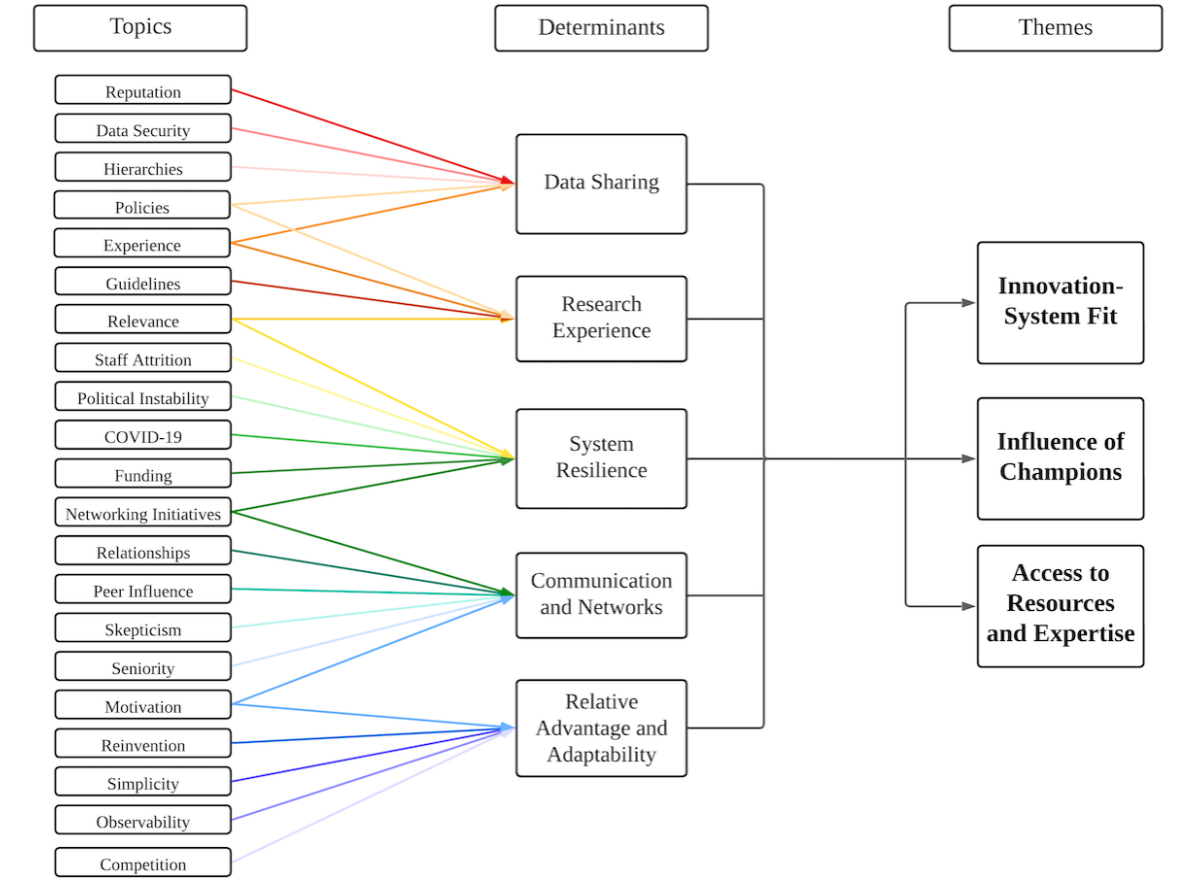
Topics, determinants, and themes of implementation.

Analysis of stakeholder interviews identified 3 key themes of implementation (Figure 3): innovation-system fit, influence of champions, and access to resources and expertise. Innovation-system fit refers to whether the registry is aligned with existing ways of working and values. Influence of champions alludes to the CLs’ and NLs’ (ie, “champions”) ability to sufficiently influence organizations to enable implementation. Finally, access to resources and expertise describes how access to software, hardware, data governance expertise, and human and financial resources was essential for implementation. These themes were born out of 21 topics (italicized throughout the results) raised in interviews and further categorized as 5 determinants presented in the remaining results.

**Table 1:** Characteristics of participants.

Characteristics		Participants, n (%)
**Sex**		
	Female	12 (37.5)
	Male	20 (62.5)
**Clinical background**		
	Yes	18 (56.3)
	No	14 (43.8)
**Time working with the registry^a^**		
	<6 months	6 (18.8)
	6-12 months	7 (21.9)
	>12 months	19 (59.4)

^a^At the time of the interview.

### Data Sharing

The implications of sharing patient data raised concerns at several levels within sites and made registry implementation harder. Department heads, concerned about the potential impact on their unit’s *reputation,* were viewed as hindering implementation by not granting necessary approvals. Clinical staff expressed fears about *data security*, particularly regarding mortality data, and occasionally prevented external DCs from entering their unit. Participants indicated that senior clinicians were concerned about data sharing because it would challenge their position within the hospital *hierarchy*. A lack of institutional *policies* and *experience* with data sharing prompted concerns by hospital management because they were uncertain how data would be managed and feared the data would belong to someone else.

Just the thought that their data is with somebody is… (Clinicians) are not comfortable with that. That’s the reason why we don’t have many registries, because people will not share data.

NL2

I have failed to come across a hospital whose policy would help us in establishing the registry. This is primarily either due to their own system being in place and they don’t want to share that data, or due to the policy not existing regarding what to do with the patients’ data.

CL5

Although data sharing concerns affected the innovation-system fit within hospitals, participants described how concerns were successfully addressed by housing data locally and emphasizing registry security features through champion-led dialogue. They also explained how the IT’s data governance expertise was leveraged to support sites establish procedures in line with local policies.

### Research Experience

Based on the experiences of NLs and ITs, implementation was easier, and adopters were more supported in hospitals where individuals had research *experience*. A lack of institutional research *policies* or *guidelines* and limited experience with existing registries additionally hampered implementation because hospital management was uncertain about registry governance. After implementation, the generation of output was hindered by uncertainty regarding the *relevance* of data for clinicians and institutions.

There are not many registries like this. So, everyone is not sure how this registry will be controlled, or who will be supervising, or who will be taking ownership of the registry.

CL3

Our head of department of anesthesia...has taken some training on patient safety and quality, informatics. He helped me, because he understood the importance of having a data set and the registry.

NL3

Although a lack of research experience decreased innovation-system fit and meant that fewer existing resources were available to national teams to support implementation, participants described how the registry’s simplicity, the influence of champions with research experience, and support from ITs helped overcome this problem.

### System Resilience

The ability of institutions to respond and adapt to changing circumstances (ie, the resilience of their system) helped and hindered registry implementation. Implementation was hampered by *political instability*. One participant explained how strikes led to doctor resignations that disrupted implementation; another related how gaining government approval was affected by unpredictable political events (see NL4 excerpt quote below). *Staff attrition* affected registry sustainability: the loss of a CL led to a case of registry abandonment, and the loss of DCs resulted in a pause in data collection.

(The site) decided to withdraw from the registry because the site director went to Australia in pursuit of his own career and the people who took over in that hospital were either not interested or not able to sustain the effort.

NL1

I booked 2 appointments with the Health Minister. But the thing is that...he was thrown out of the Ministry for no reason. Now I am looking for someone who can help us with (gaining approvals).

NL4

Participants explained how competing priorities, such as the COVID-19 pandemic, threatened registry sustainability because resources were diverted, particularly if the *relevance* of the registry was not appreciated by hospital management. However, COVID-19 also served as an impetus for decision-makers to engage in data collection, and thus, despite travel restrictions and an increased workload, the number of registry sites increased during the pandemic. *Funding* received through the CCA was essential for establishing and sustaining DCs, national, and central teams. *Networking initiatives* facilitated implementation through the sharing of learning and resources.

The registry fit better in resilient systems that can adapt to change. According to participants, resilience was built by creating a community of practice within the registry network, having champions use their influence to navigate systems, and having ITs provide human resources.

### Communication and Networks

Formal *networking initiatives* organized by ITs and preexisting professional *relationships* were cited as substantial enablers of registry diffusion and dissemination. Where an existing connection did not exist, CLs and NLs reported building personal connections with key individuals, such as department heads and opinion leaders, to be crucial to enable diffusion and dissemination. CLs and DCs explained that knowing *peers* in their country had successfully implemented the registry increased their *motivation*. Some units were *skeptical* of external individuals and international networks, which made diffusion and dissemination harder.

One CL reported that the implementation of the registry was opposed by a few *senior* clinicians because they did not want a system that would increase their accountability. *Motivated* CLs and NLs described how their *seniority* and good working *relationships* facilitated implementation. CLs and DCs emphasized how the supportive *relationship* with the ITs facilitated implementation and promoted collaboration within and outside of each country.

When we recruit another site in the same country, it usually works better that the approach has been through another clinical lead...because it’s coming from one of their colleagues.

IT1

I was fortunate to also get a chance to go to Bangkok (to the CCA project kick-off meeting). That is where my interest really picked up, that people are very motivated and they really want things to go somewhere. That was primarily the reason I started pushing (the registry) here.

CL1

### Relative Advantage and Adaptability

Participants viewed the ability to *reinvent* the registry—to adapt it to varied contexts—to be important in helping with implementation. The registry could be used for different purposes, including pandemic surveillance, quality improvement, and trials, as well as refined to remove or rename variables to reflect local practice. Registry *reinvention* was prioritized by the DT, which acknowledged that keeping up with demands was challenging but vital for implementation and user confidence.

When we have something that the collaborators need, I think it’s better to do it because if we stop that thing (from being done), they will stop the data collection and also struggle with those things.

DT2

The *simplicity* of the registry was another important feature that helped with implementation. CLs and DCs described how it did not require specialist technical or medical knowledge to operate, and reported that registry data were more comprehensive and simpler to access than paper records. The *observable* value of the registry, in terms of publications and professional development opportunities, further *motivated* champions and encouraged new sites to join.

We have a responsive system, which I like to think is a living platform. I think it’s perhaps the most important (factor), that it’s a platform, which can adapt and evolve.

IT3

Previously we didn't have any records. We would transfer everything into the medical records department and we would not get it back. But now we are collecting it, we are maintaining it and I think this will help us in many ways.

DC2

Participants explained how the registry was advantageous for government health policies and the priorities of professional bodies. Unfortunately, engagement with them was not always conducive. One commonly cited reason for this was that the registry was seen as *competition* because it either directly competed with an existing or planned project or undermined the authority of the government or professional organization.

## Discussion

This study describes stakeholder perspectives on the implementation of a critical care registry in units across 4 countries in South Asia. Examining registry implementation highlighted 3 key themes: innovation-system fit, influence of champions, and access to resources and expertise. Implementation, therefore, was possible in settings where the registry aligned with usual ways of working, where a champion was present to promote implementation, and where those implementing the registry had access to expertise and technical, human, and financial resources. These resonate with findings from other research and expert commentaries on digital health innovation implementation [12,13,18].

Innovation-system fit was promoted by the simple, user-friendly, and adaptable nature of the registry, as well as its relative advantage over previous ways of working, attributes that have been consistently shown to increase the likelihood of implementation [12-14]. Although the registry itself was a relatively simple technical innovation, it brought with it notions of data sharing and research that interfered with existing values, thus reducing system fit. This lack of fit was exemplified by reluctance to share data in the context of nascent institutional data sharing and research policies. These realities in the implementation settings and beyond will hamper the operationalization of registries as well as high-quality health systems, which depend on research literacy and supportive governance structures [1,2,18,23]. A few individuals were skeptical about the registry, viewing it as an outside agenda, which also reduced innovation-system fit. This is not new; previous implementation studies have shown a lack of ownership, user engagement, and acceptance of systems seen to be implemented by “outsiders” or in a top-down manner, such as government initiatives with a large scope and mandate [14,16,17,30]. However, the CCA registry does not have a centrally driven mandate for sites. Instead, it provides an adaptable registry for stakeholders to pursue their priorities, ensuring better innovation-system fit.

Champions, motivated to pursue their priorities with the registry, played a key role in addressing concerns about innovation-system fit by encouraging well-connected clinicians to take ownership of the registry. In fact, a champion-led approach was essential throughout implementation because these individuals built networks to promote dissemination, worked to overcome bureaucratic and institutional barriers preventing adoption, and navigated hurdles threatening sustainability. Our study reinforces and exemplifies the importance of local leadership and ownership and the role of champions in enabling implementation, as highlighted by many others [12,14,16]. However, champions are not the only actors that influence the implementation of innovations in health care. Thus, a champion-led approach does not remove competing priorities and tensions among different groups, such as government and professional bodies [17,31]. Without reconciliation of these tensions, the sustainability of the registry is at risk, as these groups have substantial influence on system resilience [3].

Access to resources, expertise, and peer support through the CCA community of practice was vital in enabling the champion-led approach to implementing the registry. Collaboration provided encouragement and motivation to individuals, promoted system resilience, and supplied resources that were otherwise unavailable to stakeholders [3]. However, reliance on the CCA for funding and other resources raises questions about sustainability, particularly in the face of challenges such as political instability, staff attrition, and pandemics [32]. Additionally, reliance on a few champions does pose questions regarding sustainability, scalability, and representativeness. Nonetheless, expansion to new sites despite the COVID-19 pandemic is a reason for optimism.

### Strengths and Limitations

This study’s strengths include the use of a theory-based analytical framework for evaluation and the inclusion of stakeholders with varied lengths of time participating in the registry [12]. A key limitation was the absence of nonadopters, those who abandoned the registry, and representatives of government and professional bodies in the interviews. Dissenting perspectives could have provided additional insight and represents a valuable avenue for further inquiry [31]. However, this study aimed to understand the perspectives of those that completed implementation to understand context-relevant determinants of implementation, which help current stakeholders in registry implementation. The convenience sample of sites additionally introduced bias; however, the impact is suspected to be minimal as included sites were diverse in geographical location, size, and unit type. Lastly, high-quality health systems require data not only to be collected but also analyzed and used to inform cycles of learning [1,2]. This aspect was not fully explored in our study, as CCA stakeholders were focused mainly on implementation at the time of the interviews. Registry long-term sustainability and use of data should therefore be explored in subsequent evaluations.

### Conclusions

The CCA registry has been implemented to support the development of high-quality health systems in Asia. Implementation has been possible because the registry can be adapted to fit the systems in which LMIC clinicians work, and implementation was enabled by the influence of motivated champions, shared expertise, and access to additional resources. In view of reliance on individuals and competing priorities of other health care actors, questions about sustainability remain and will be explored further in future research.

## Appendix

Multimedia Appendix 1 Supplementary information and tables.

## Abbreviations

CCA: Collaboration for Research, Implementation, and Training in Critical Care in Asia
CL: clinical lead
DC: data collector
DT: development team
IRIS: Indian Registry of Intensive Care
IT: implementation team
LMIC: low- and middle-income country
NICRF: Nepal Intensive Care Registry Foundation
NL: national lead
PRICE: Pakistan Registry of Intensive Care

## Data Availability

The interview guide for this study is provided in Multimedia Appendix 1. Interview recordings will not be available in order to protect participant confidentiality. All deidentified participant quotes supporting the results of this study will be made available on request to the corresponding author. For the purpose of Open Access, we have applied a CC-BY public copyright license to any Author Accepted Manuscript version arising from this submission.
