# Supplementary material for: Determinants of Implementation of a Critical Care Registry in Asia: Lessons From a Qualitative Study
Source: J Med Internet Res. 2023 Mar 6;25:e41028. doi: 10.2196/41028 (PMC10028509; doi:10.2196/41028)
Supplement: Multimedia Appendix 1 [file jmir_v25i1e41028_app1.pdf]

Multimedia Appendix 1

This is a Multimedia Appendix to a full manuscript published in the J Med Internet Res titled 'Determinants of implementation of a critical care registry in Asia: lessons from a qualitative study'. For full copyright and citation information see http://dx.doi.org/10.2196/jmir.41028

Table of contents

1. Collaboration of Research Implementation and Training in Critical Care in Asia Investigators 2

2. Characteristics of sites and clinical registries included in the study 4

Table 2.1. Characteristics of sites and intensive care units (ICUs) represented in this study 4

Table 2.2. Characteristics of clinical registries of the Collaboration for Research Improvement and Training in Critical Care in Asia (CCA) network included in the study at the time of the interviews 5

3. Template for Intervention Description and Replication (TIDieR) checklist 6

4. Standards for Reporting Qualitative Research Checklist 10

5. Interview guide 12

1. Collaboration of Research Implementation and Training in Critical Care in Asia Investigators

Writing group
Timo Tolppa, Vrindha Pari, Christopher Pell, Diptesh Aryal, Madiha Hashmi, Maryam Shamal Ghalib, Issrah Jawad, Swagata Tripathy, Bharath Kumar Tirupakuzhi Vijayaraghavan, Abi Beane, Arjen M Dondorp and Rashan Haniffa.

Collaborators

AFGHANISTAN (Registry for Intensive Care in Afghanistan - RICA)
Maryam Shamal Ghalib (Country Coordinator), Ahmad Zekria Sherzai (Data Validator), Ahmad Seyar Quraishi (Jamhoriat Tertiary Hospital, Kabul), Meharnegar Haqyar Mohammadi (Wazir Akbar Khan Hospital, Kabul), Ghulam Rahim Awab, Noorullah Ahmadzai, Guldad Khan Safi (Nangarhar Regional Hospital, Jalalabad), Mirwais Azizi (Ali Jenah Hospital, Kabul), Asilah Hedayat (Herat Regional Hospital, Herat); Naseebullah Barekzai, Dawood Safi (Indira Gandhi Children's Hospital, Kabul), Dr Zabihullah Adeeb (Ariana Medical Complex, Kabul), Dr Zakia Kohistani (Malalai Maternity Hospital, Kabul), Asia Shamal (Shefajo Hospital, Kabul), Hamid Safi (Sheikh Zayed Hospital, Kabul).

INDIA (Indian Registry of IntenSive Care - IRIS)
Pratheema Ramachandran, Suresh Babu Masilamani (Apollo Speciality Hospital, OMR, Chennai); Bharath Kumar Tirupakuzhi Vijayaraghavan, Augustian James, Nagarajan Ramakrishnan, Ramesh Venkataraman, Lakshmi Ranganathan (Apollo Main Hospital, Chennai); Meghena Mathew, Revathi Kandeepan (Apollo First Med Hospital, Chennai); Ebenezer Rabindrarajan, Madhu Shree, Usha Rani Chandramohan (Apollo Speciality Hospital, Vanagaram); Jaganathan Selvanayagam, Thirumalai Sambath (Mehta Hospital, Chennai); Mathew Pulicken, Ashley Aniyakunj (Pushpagiri Medical College Hospital, Kerala); Rakesh Lakshmappa, Karthik Shivani Lokeshappa (Nanjappa Multispecialty Hospital, Karnataka); Raymond Savio, Sristi Patodia, Premnath Balakrishnan (Apollo Proton Cancer Center); Kishore Mangal, Disha Chandel (Eternal Hospital, Jaipur Rajasthan); Deepak Vijayan, Krishna Priya, (KIMS, Kerala); Rajyabardhan Patnaik, Kasi Chinni Krishna (ISPAT General Hospital, Rourkela); Kavita Kamineni, Saradha Chirravuri (ABC Hospital, Visakhapatnam); Swagata Tripathy, Kasturi Sanyal (AIIMS, Bhubaneswar); Zubair Mohamed, Anna Paul (Amrita Institute of Medical Sciences, Kerala), Atul Kulkarni, Anjana Shrivastava, Nikita Kulaye, Sneha Gupta (Tata Memorial Hospital, Mumbai), Devachandran Jayakumar, Ajith Samy (Dr. Kamakshi Memorial Hospital, Chennai), Darshana Rathod, Minal Shah (Sir HN Reliance Foundation Hospital, Mumbai), Balaji Venkatachalam, Bharath Kumar Krishnarao (Vijaya Medical and Educational Trust, Chennai), Urvi Shukla, Chinmayee Bhise (Symbiosis University Hospital and Research Centre, Pune).

NEPAL (Nepal Intensive Care Registry Foundation - NICRF)
Kishor Khanal, Ashim Regmi, Namrata Rai, Kanchan Koirala, Kaveri Thapa, Krisha Dheke, Manisha Maharjan (Nepal Mediciti Hospital, Lalitpur); Subhash Prasad Acharya, Kabita Sitoula, Asmita Pokhrel, Namrata Shrestha, Saraswoti Sharma, Bimala Make, Arati Phuyal, Radhika Maharjan, Sabi Bajracharya, Roshni Thapa, Binita Bhattarai (Tribhuvan University Teaching Hospital, Kathmandu); Diptesh Aryal, Sabin Koirala, Hem Raj Paneru, Sujata Chauhan, Angela Lamichhane, Alina Lamichhane, Sangita GC, Swastika Phuyal, Crystal Maharjan, Anusha Subedi, Bini Kayastha, Kabita Khadka, Rakshya Karki, Roshani Shakya, Srijana Kayastha, Pratibha Paudel (Hospital for Advanced Medicine and Surgery HAMS, Kathmandu); Sushil Khanal, Samina Amatya, Shreya Pathak, Pujan Rajbhandary, Bina Bhattarai, Sabita Shrestha, Sharmila Mali (Grande International Hospital); Shital Adhikari, Basanta Gauli, Nisha Bhandari, Babita Ghimire, Asmita Kaini (Chitwan Medical College, Chitwan); Pramesh Shrestha, Bipin Karki, Roshni Karki, Sabina Dhakal, Mandira Thapa, Sarita Tamang (Om Hospital, Kathmandu); Shubha Kalyan Shrestha, Roshni Kafle, Kalpana Gurung, Sheela Shrestha (Karuna Hospital, Kathmandu); Sanjay Lakhey, Tamanna Bajracharya, Anita Bashya, Prajina Malla, Jeeya Deuja (B&B Hospital, Lalitpur); Anand Thakur, Santosh Acharya, Radhika Maharjan, Sachita Maharjan, Subina Maharjan Yashu (Nidan Hospital, Lalitpur); Raju Shrestha, Pratima Sigdel, Merina Pradhan (B & C Hospital), Pradip Tiwari, Rajani Shrestha, Renu Younjan (Civil Service Hospital, Kathmandu); Lalit Rajbanshi, Nanu Waiba, Rita Das (Birat Medical College); Subekshya Luitel, Shirish KC (NICRF, Kathmandu).

PAKISTAN (Pakistan Registry of Intensive Care - PRICE)
Madiha Hashmi, Ashok Kumar, Mukesh Kumar, Quratul Ain Khan, Osama Khalid, Ali Raza, Ali Abbas, Akash Thakrani, Adnan Gul, Ilyas Shehzad, Samad Ali, Mohiuddin Shaikh, Faheem Shaikh, Fasiha Khan, Suamira Qabulio, Ahmed Khan (Ziauddin Group of Hospitals, Karachi); Dr Attaur Rehman, Amir Khan, Ahmed Zia, Farhan Khan (Patel Hospital, Karachi); Nawal Salahuddin, Amin Khawaja, Mohammad Imran, Vinod Kumar (National Institute of Cardiovascular Diseases, Karachi); Tanvir Alam (Civil Hospital, Karachi); Nadeem Muneer, Vinod Kumar (Jinnah Post-Graduate Medical Center, Karachi); Aneela Altaf Kidwai, Aftab Ahmed, Faizan Khan, Mohammad Naveed (Abbasi Shaheed Hospital, Karachi); Rashid Nasim Khan, Jhonsan Shahzad (Darul Sehat Hospital, Karachi); Saeeda Haider, Fivzia Herekar, Arther John, Yasir Rehman (The Indus Hospital, Karachi); Fakhir Raza Haidri, Fawadur Rehman (SIUT, Karachi); Muhammad Nasir Khoso, Ahmed Khan, Huda Siddiqui (South City Hospital, Karachi); Saleh Khaskheli, Muhammad Ibrahim (Peoples Medical University, Nawabshah); Kashif Memon, Mohsin Ali (Liaquat University Hospital, Hyderabad); Maqsood Meher, Itefaq Ahmed (GMMM Teaching Hospital, Sukkur); Sayed Muneeb Ali, Rana Imran Sikandar, Imtiaz Ali Shah (Pakistan Institute of Medical Sciences, Islamabad); Liaquat Ali, Muhammad

Ashraf Zia, Moazzam Tarar, Ahmed Ranjha, Basit Ali, Shahryar Maqsood (Jinnah Hospital, Lahore); Ahmed Farooq, Ehtisham Waheed (Doctors Hospital, Lahore); Arshad Taqi, Dr. Hashim Ghouri (National Hospital & Medical Center, Lahore); Jodat Saleem, Afia Arshad, Irfan malik, Rehan Niazi, Shahryar Maqsood (Lahore General Hospital, Lahore); Naseem Ali Shah (Hameed Latif Hospital, Lahore); Kamran Cheema, Mazhar Ali Naqvi, Basit Ali (Services Hospital, Lahore); Iqbal Hussain, Mobin Chaudhary, Sadia Ghulam Mustafa (Pakistan Kidney and Liver Institute, Lahore); Sairah Sadaf, Nayyara Sameen (Sheikh Zayed Medical College, Rahim Yar Khan); Muhammad Hayat, Arslan Rahatullah, Muhammad Kamran, Farman Ali Khan, Ihtisham Ali (North West General Hospital, Peshawar); Muhammad Sheharyar, Sajjad Orakzai, Zafar Iqbal Khatak (Lady Reading Hospital, Peshawar); Imran ul Haq, Farman Ali Khan (Khyber Teaching Hospital, Peshawar); Musteshan Bashir, Anum Akram, Omar Nazeer (Mayo Hospital, Lahore); Shereen Khan, Syed Hidayat Ullah, Ali Asghar (Fatima Jinnah Chest Institute, Quetta).

Central Coordination, Development and Implementation Team
Udara Attanayake, Abi Beane, Sri Darshana, Arjen M Dondorp, Layoni Dullewe, Nilmini P Dullewe, Kaumali Gimhani, Rashan Haniffa, Pramodya Ishani, Chamira Kodippily, Issrah Jawad, Himasha Muvindi, Luigi Pisani, Dilanthi Priyadarshani, Disna Pujika, Aasiyah Rashan, Sumayyah Rashan, Thalha Rashan, Timo Tolppa, Ishara Udayanga.

2. Characteristics of sites and clinical registries included in the study

Table 2.1. Characteristics of sites and intensive care units (ICUs) represented in this study

Site	Country	State or Province	Type of ICU[a]	Number of ICU beds	Length of time participating in registry (months)[b]
Site 1	India	Tamil Nadu	Mixed ICU	24	19
Site 2	India	Odisha	Mixed ICU	12	10
Site 3	India	Tamil Nadu	Mixed ICU	19	19
Site 4	India	Kerala	Mixed ICU, Other[c]	41	2
Site 5	Nepal	Bagmati	Mixed ICU	33	11
Site 6	Nepal	Bagmati	Mixed ICU	21	12
Site 7	Nepal	Bagmati	Mixed ICU	30	8
Site 8	Nepal	Bagmati	Mixed ICU	11	12
Site 9	Pakistan	Sindh	MICU, SARI ICU, SICU	27	21
Site 10	Pakistan	Sindh	MICU, SARI ICU, SICU	42	24
Site 11	Pakistan	Sindh	SARI ICU	14	5
Site 12	Pakistan	Khyber Pakhtunkhwa	HDU, MICU, Mixed ICU, SARI ICU, SICU	38	24
Site 13	Pakistan	Islamabad Capital Territory	MICU, SARI ICU, SICU	29	24
Site 14	Afghanistan	Kabul	MICU, Mixed ICU	24	7
Site 15	Afghanistan	Nangarhar	MICU, Mixed ICU	26	4

[a]HDU, high dependency unit; MICU, medical ICU; SARI, severe acute respiratory infection; SICU, surgical ICU
[b]At the time of the interviews
[c]Includes emergency, neurology, head & neck, and liver transplant ICUs

Table 2.2. Characteristics of clinical registries of the Collaboration for Research Improvement and Training in Critical Care in Asia (CCA) network included in the study at the time of the interviews

Adapted from Collaboration for Research, Implementation and Training in Critical Care - Asia Investigators et al [8].

Characteristic	Afghanistan	India	Nepal	Pakistan	Total
Patient episodes[a]	553	4,675	2,951	10,972	19,151
Number of ICUs	6	18	8	55	87
Number of beds	60	213	138	557	968
Type of ICUs[b]					
Mixed ICU	5	13	6	7	31
MICU	1	1	1	12	15
SICU				19	19
CT ICU				1	1
SARI ICU		1	1	13	15
HDU				1	1
Other		3		2	2

[a]During the six month period of June to December 2020
[b]CT ICU, cardio-thoracic ICU; HDU, high dependency unit; MICU, medical ICU; SARI, severe acute respiratory infection; SICU, surgical ICU

3. Template for Intervention Description and Replication (TIDieR) checklist [24]

TIDieR Item	TIDieR Item Guidance	CCA Registry Description
1. Brief name	Provide the name or a phrase that describes the intervention.	To implement a cloud-based setting-adapted registry platform in 42 intensive care units in nine low-income and middle-income countries in Asia.
2. Why	Describe any rationale, theory, or goal of the elements essential to the intervention.	To provide real-time data on service activity, case mix, processes of care, patient experience and outcomes for critical care services in Asia.
3. What - Materials	Describe any physical or informational materials used in the intervention, including those provided to participants or used in intervention delivery or in training of intervention providers. Provide information on where the materials can be accessed (such as online appendix, URL).	Materials required for implementation included:<br>● Invitation letter<br>● CCA project information sheet<br>● Research collaboration agreement<br>● Web-based registry portal<br>● Landscaping survey<br>● Institutional and ethical application templates<br>● Registry data structure diagram<br>● Data collection guides<br>● Data dictionary<br>● Guidance for validation and data quality assurance<br>● Good Clinical Practice eLearning courses
4. What - Procedures	Describe each of the procedures, activities, and/or processes used in the intervention, including any enabling or support activities	Networking<br>- Attendance and presentation at national and international conferences<br>- New collaborators reaching out over social media or in response to publications<br>- Contact through existing personal and professional contacts<br>- Inaugural CCA network project meeting<br>- National registry networking meetings<br><br>Establishment of a new registry collaboration<br>- Appointment of national clinical lead and national implementation coordinator<br>- National lead training and internship with established registries in the network<br>- Weekly conference calls between national leads and NICS-MORU (National Intensive Care Surveillance-Mahidol Oxford Tropical Medicine Research Unit) team<br>- Monthly meetings with national implementation coordinators<br>- Engagement with professional bodies including critical care societies, regional training programmes, academic institutions and the Ministry of Health for support and input<br>- Review of ethics requirements and waiver or application for approval, where appropriate<br>- Provision of technical support for the registry, data security management, visualisation, analysis and storage<br>- Signing of a memorandum of understanding<br><br>Establishment of a new site (Implementation)<br>- Recruitment of local clinical lead<br>- Demonstration session introducing the registry via a shared screen<br>- Trial of data entry on registry with unique user details<br>- Review of institutional ethics requirements and waiver or application for approval<br>- Procurement of necessary hospital management approvals<br>- Completion of ICU landscaping survey to find out about unit facilities, equipment, staffing, guidelines, checklists and units of measure for clinical and laboratory variables<br>- Appointment of data collectors<br>- Data collector training onsite or remotely via videoconferencing<br>- Provision or guidance on acquiring 3G connection or wifi, and devices

		- Review of server housing and decision-making on housing server locally or in Sri Lanka<br>- Daily messaging and telephone contact from the coordinating NICS-MORU team<br>- Frontline ICU healthcare teams and clinical stakeholder (doctors, nurses) training in registry navigation, desktop application and dashboard use<br>- Online Good Clinical Practice training<br>- Telephone support and app-based messaging groups to provide technical support<br><br>Post-Implementation Support<br>- Weekly or fortnightly review of data completeness, frequency of reporting and validity of data entry with the national and NICS-MORU implementation teams<br>- Data validation queries from automated and manual checks sent to sites<br>- Errors, edits and validation queries generated are stored separately, so that common errors could be identified and acted on<br>- Adaptation of the registry where necessary<br>- E.g. variable labels were adapted to reflect local healthcare system terminology, user interfaces were adapted to maximise data completeness and field validation or alerts were added to reduce user error.<br>- E.g. data fields added and/or edited due to local projects and research questions or participation in observational studies, pandemic surveillance, international trials or other data collection<br>- Creation and testing of new developments to the registry<br>- Training and close communication following the release of new registry features<br>- Constant technical and operational support through telephone support and app-based messaging groups, and meetings as required by sites<br>- Production of customised reports and analyses of data based on site requirements
5. Who provided	For each category of intervention provider (such as psychologist, nursing assistant), describe their expertise, background, and any specific training given for the implementation.	NICS-MORU implementation team<br>- *Project coordinators:* Overall responsibility for the design of the registry, implementation and strategic vision for the network. Involved in coordinating the development of the registry, identifying potential collaborators, supporting stakeholders in implementation and ensuring sustainability through ensuring utility of the registry, demonstrating the value to stakeholders as well as potential research partners and funders.<br>- *Implementation coordinators:* The NICS-MORU implementation coordinators' centrally managed operational aspects related to the implementation of the registry. They assisted national coordinators in all phases of the project start-up, approval submissions, implementation and data monitoring, and coordinated between the local, national and development teams. They assisted national and local coordinators in ensuring data safety and quality, and provided post-implementation support.<br><br>NICS-MORU development team<br>- *Data analyst:* Provided technical support for the registry and data visualization.<br>- *Registry developer:* Manage software development and adaptation of the registry.<br>- *Database manager:* Maintenance, management and curation of the registry database.<br>- *Biostatistician:* Performed the statistical analyses of the quantitative and qualitative data from observational and quality improvement activities.<br>- *Visualisation developer:* Developed real-time data visualisation for individual sites ausing customised dashboards supported by human-computer interaction theory.<br><br>National implementation teams<br>- *National leads:* These individuals acted as liaison between the NICS-MORU teams and the sites in the country. They helped identify sites, clinical leads and data collectors within each country, held an advocacy role and acted as a liaison for clinical, professional and ministerial stakeholders. They ensured that all local necessary ethical and regulatory approvals were obtained before the start of implementation.<br>- *Implementation coordinators:* Under the supervision of the NICS-MORU implementation coordinators, the implementation coordinators supported sites in their country with logistics, administration, implementation and post-implementation support.<br><br>Site teams<br>- *Clinical leads:* In each site, a clinical lead (doctor or senior nurse) was appointed, who led local implementation, acquisition of necessary approvals, supported peer training and troubleshooted problems in the daily use of the registry as well as guided the local use of the data. They reported to the national and NICS-MORU implementation teams.

		- *Data collectors:* Data collectors, either clinical or non-clinical depending on site preferences, were appointed in each unit and trained in data entry and dashboard navigation.
6. How	Describe the modes of delivery (such as face to face or by some other mechanism, such as internet or telephone) of the intervention and whether it is provided individually or in a group	● Face to face: Members of the NICS-MORU implementation team visited new sites to deliver training and collect feedback from stakeholders directly.<br>● Video conferencing: Shared screen video conferencing such as Skype, Hangouts and Zoom were used to deliver training on data input and validation on the platform individually and to groups. Training for new platform features was also conducted using video conferencing.<br>● Instant messaging: Web-based 24 hr implementation and technical support was available via WhatsApp in all countries.<br>● Email: Email addresses of each country's team members and the central team were shared and email threads saved as records of discussions.<br>● Via the registry portal: The reporting facilities provided near real-time reporting on the data quality including completeness, epidemiology, severity of illness, treatment, microbiology and outcomes of ICU patients.
7. Where	Describe the type(s) of location(s) where the intervention occurred, including any necessary infrastructure for relevant features.	The registry implementation occured on-site in all the relevant units and was centrally overseen by NICS-MORU based in Sri Lanka. Infrastructure required for implementation has been outlined in section 4 (implementation) in detail and included:<br>● 3G connection or wifi<br>● Mobile, desktop or tablet<br>● A physical server, housed in a secure location at the network site country or at NICS-MORU's secure location.
8. When and how much	Describe the number of times the intervention was delivered and over what period of time including the number of sessions, their schedule,and their duration, intensity, or dose	● Initial training and set up: This was a period of close supervision, communication and support including email, web-based 24 hour implementation and technical support available via WhatsApp, training and meetings to discuss any issues.<br>● Support: Weekly video conferencing sessions with sites and implementation teams provided an opportunity for troubleshooting and feedback on feasibility, usability and enable adaptation to be discussed and prioritised.<br>● Feedback and iteration: Twenty-four hour support via online messaging was available during implementation and post-implementation support.
9. Tailoring	If the intervention was planned to be personalised, titrated or adapted, then describe what, why, when, and how	The steps that were undertaken to ensure tailoring of the platform to each site have been outlined in detail in Section 4. All iterations were guided by stakeholders, led by the implementation team and conducted by the development team. Iterations included the following:<br>● Landscaping survey to tailor the data set and the platform identifiers - number of beds, unit names, logos, person identifiers, users and logins.<br>● The data dictionaries were produced collaboratively and local terminology as well as preferences for units of measure and bioclinical information were taken at the start.<br>● Network countries had a choice of optional forms for quality indicators and electronic health record features including daily observations and investigations.
10. Modifications	If the intervention was modified during the course the study, describe the changes (what,why,when,and how)	● Weekly video conferencing sessions with sites and implementation teams provided an opportunity for feedback on feasibility, usability and enabled adaptation to be discussed and prioritised.<br>● Twenty-four hour support via online messaging was available during implementation and post-implementation. An iterative cycle of adaptation and implementation was used to adapt the registry where necessary.<br>● Examples of areas of modifications included:<br>○ *Login and access portals*<br>○ *Data set* - core data set, ability to embed additional variables for research studies. Ability to embed quality improvement audits. Ability to report pandemic surveillance.<br>○ *User interface:* Navigation changes<br>○ *Visualisation and output:* Stakeholders identified which indicators or outputs they wanted prioritised including occupancy, acuity, equipment and resource availability and bed capacity<br>○ *Reports and PDF generation:* Modifications were made to what variables and indicators were reported in the unit and registry reports.<br>○ *Field labels:* These were adapted to reflect local healthcare system terminology, user interfaces were adapted to maximise data completeness and field validation and alerts were added in a personalised way to reduce user error.

11. How well - Planned	If intervention adherence or fidelity is assessed, describe how and by whom, and if any strategies were used to maintain improve fidelity, describe them	Adherence or fidelity in the context of implementing the CCA registry refers to the extent of achieving the target of 42 units in nine South Asian countries over three years. This is monitored by the NICS-MORU implementation team and reviewed in regular project grant meetings. Recruitment of additional units and countries proceeded as described in Section 4.<br><br>Fidelity and accuracy of data capture for the registry was assessed and supported using the strategies described below. Further information about these processes is available in Section 4.<br>- Data validation comprised automated rules within the registry, on-site checks and remote checks by the NICS-MORU team. Automated rules flagged abnormally high or low values and missed information. Use of free text entry was restricted as much as possible.<br>- Visual display of daily data capture facilitated checks on accuracy and completeness by the clinical lead and data collector. Checks were subsequently also performed centrally by the NICS-MORU team, who monitored data entry daily, aided by automated scripts.<br>- Queries were sent back to the sites, which were addressed by the data collector and clinical lead, and overseen by the national coordinator.<br>- Errors, edits and validation queries generated in the registry were stored separately. This allowed common errors to be identified and acted on.<br>- Rapid feedback loops were maintained, since in the low-income and middle-income setting traceability of paper-based notes is generally limited. The use of a single platform for entering, querying, reporting and validating data increased efficiencies.
12. How well - Actual	If intervention adherence or fidelity was assessed, describe the extent to which the intervention was delivered as planned	The achievement of the work packages of the CCA registry are monitored annually by the NICS-MORU project coordinators and reported to the grant committee.<br><br>Data reporting from the registry is as follows:<br><br>● Private reporting<br>All sites had immediate access to their own unit's information captured through the registry via the private dashboard. This information enabled healthcare staff, administrators and researchers to evaluate trends in unit activity, severity of illness, bed occupancy, length of stay and outcomes within their respective institution.<br><br>● Peer reporting<br>The aim of peer reporting was to enable evaluation of performance between comparable units. Benchmarking was used to enable stakeholders within the network to evaluate services, identify 'good or effective practice' and share this with other units. Benchmarking processes of care and outcomes helped healthcare leaders identify relationships between resource, infrastructure, healthcare culture and experience, complimenting the qualitative health systems evaluation which was undertaken by the network and the training to support stakeholders to accurately evaluate and interpret the indicators of quality measured through the registry.<br><br>● Public reporting<br>Anonymised aggregate data dashboards were accessible through a public portal. The aim was to provide community stakeholders with the opportunity to see information about healthcare within their region or country, promoting greater accountability of care. The dashboards were tailored to the patient and public priorities and included supportive information to aid interpretation. The information was used to inform those responsible for strategic planning and financial provision of healthcare at regional and national level.

4. Standards for Reporting Qualitative Research Checklist [26]

No	Topic	Item	Location where item reported
**Title and abstract**			
S1	Title	Concise description of the nature and topic of the study identifying the study as qualitative or indicating the approach (e.g., ethnography, grounded theory) or data collection methods (e.g., interview, focus group) is recommended.	1
S2	Abstract	Summary of key elements of the study using the abstract format of the intended publication; typically includes background, purpose, methods, results, and conclusions.	1
**Introduction**			
S3	Problem formulation	Description and significance of the problem/phenomenon studied; review of relevant theory and empirical work; problem statement.	2-3
S4	Purpose or research question	Purpose of the study and specific objectives or questions.	3
**Methods**			
S5	Qualitative approach and research paradigm	Qualitative approach (e.g., ethnography, grounded theory, case study, phenomenology, narrative research) and guiding theory if appropriate; identifying the research paradigm (e.g., postpositivist, constructivist/interpretivist) is also recommended.	3
S6	Researcher characteristics and reflexivity	Researchers' characteristics that may influence the research, including personal attributes, qualifications/experience, relationship with participants, assumptions, and/or presuppositions; potential or actual interaction between researchers' characteristics and the research questions, approach, methods, results, and/or transferability.	4-5
S7	Context	Setting/site and salient contextual factors.	3-4<br>Appendix pp 2-3
S8	Sampling strategy	How and why research participants, documents, or events were selected; criteria for deciding when no further sampling was necessary (e.g., sampling saturation).	4
S9	Ethical issues pertaining to human subjects	Documentation of approval by an appropriate ethics review board and participant consent, or explanation for lack thereof; other confidentiality and data security issues	4
S10	Data collection methods	Types of data collected; details of data collection procedures including (as appropriate) start and stop dates of data collection and analysis, iterative process, triangulation of sources/methods, and modification of procedures in response to evolving study findings.	4
S11	Data collection instruments and technologies	Description of instruments (e.g., interview guides, questionnaires) and devices (e.g., audio recorders) used for data collection; if/how the instrument(s) changed over the course of the study.	4<br>Appendix pp 15-18
S12	Units of study	Number and relevant characteristics of participants, documents, or events included in the study; level of participation (could be reported in results).	4-5<br>Appendix pp 2-3
S13	Data processing	Methods for processing data prior to and during analysis, including transcription, data entry, data management and security, verification of data integrity, data coding, and anonymization/deidentification of excerpts.	4-5
S14	Data analysis	Process by which inferences, themes, etc., were identified and developed, including the researchers involved in data analysis; usually references a specific paradigm or approach.	5

S15	Techniques to enhance trustworthiness	Techniques to enhance trustworthiness and credibility of data analysis (e.g., member checking, audit trail, triangulation).	5
**Results/findings**			
S16	Synthesis and interpretation	Main findings (e.g., interpretations, inferences, and themes); might include development of a theory or model, or integration with prior research or theory.	5-8
S17	Links to empirical data	Evidence (e.g., quotes, field notes, text excerpts, photographs) to substantiate analytic findings	6-7
**Discussion**			
S18	Integration with prior work, implications, transferability, and contribution(s) to the field	Short summary of main findings; explanation of how findings and conclusions connect to, support, elaborate on, or challenge conclusions of earlier scholarship; discussion of scope of application/generalizability; identification of unique contribution(s) to scholarship in a discipline or field.	8-9
S19	Limitations	Trustworthiness and limitations of findings	9
**Other**			
S20	Conflicts of interest	Potential sources of influence or perceived influence on study conduct and conclusions; how these were managed.	9
S21	Funding	Sources of funding and other support; role of funders in data collection, interpretation, and reporting	1, 5

5. Interview guide

Introduction and Informed Consent
Introduce yourself and commence audio recording once the participant agrees to this. Explain the Participant Information Sheet to the participant before the interview and answer any questions they may have. Ask these questions after the explanation to confirm participation:

- Do you agree to take part and give your permission for me to audio record you?

**Theoretical domain**	**Interview questions**	**Examples/Prompts**
Role orientation	1. What is the capacity of involvement in terms of the registry? (Description of roles and responsibilities as part of the implementation)	All: Tasks performed as part of the role, dynamics with the people you are supervising or reporting to.<br><br>Use the table from 'Participant Information Sheet' as a prompt.
The attributes of the innovation	2. What are the technical features of the registry that are important for its usability?	e.g. Syncing, offline function, mobile version, reports, dashboards, CT SNOMED search function.<br><br>All: How have you used the data from the registry? How did you learn how to use the registry and platform, and how difficult was this? What made it difficult or easy? What changes to the registry have improved it since you started?<br><br>Data collectors/implementation coordinators: What specific functions make data collection/implementation easier? Has using a single platform increased efficiency? How has data visualisation affected data accuracy and completeness?
The adoption process as engaged in (or not) by individuals.<br><br>Communication and influence.	3. Can you go over what the implementation process of the registry was like at your site/at one of the sites? What did the implementation process involve?<br><br>4. How is the communication with the implementation team (includes NICS-MORU, national registry and sites) and their contribution impact implementation?	Anything about the implementation process that was particularly useful or not? e.g. training, whatsapp group, test server, feedback loops, making changes to registry<br><br>Pakistan/Nepal: Could you describe how the new SARI (COVID-19) form was introduced and implemented?<br><br>Data collectors/Implementation coordinators: Online conference training, process of incorporating suggestions to the platform. Who do you report complaints/problems to? What is your experience of this?
The inner (organisational) context (including structural determinants of innovativeness, receptive context for change in general, absorptive capacity for new knowledge, and tension for a particular change)	5. What hospital or management (organisational) factors impacted on the registry's implementation?<br><br>6. What social factors impacted on the registry's implementation?	National leads/implementation team: Was implementation easier or harder at particular hospitals? Why? Any differences between implementation in public and private hospitals?<br><br>Clinical leads: Does your organisation adopt new innovation or support quality improvement? Does the hospital have a policy which supported/hindered implementation? How was the ethical waiver or application process?<br><br>All: How was leadership during implementation? What resources have you needed for implementation? Have these been available?<br><br>e.g. Support from peers/colleagues.<br><br>All: Any individuals that played an important role in enabling or preventing implementation? How was the registry understood by others?<br><br>Hospital leads/Implementation coordinators: Has funding for data collectors helped with implementation?<br><br>Clinical leads/data collectors: How was the decision made to implement the registry? What has influenced your desire to implement the registry?

		Data collectors: Were clinical staff approachable?
The outer (extra-organisational) context (including inter-organisational collaboration and networking, prevailing environmental pressures such as external competition, particular policymaking contexts and streams, and proactive linkage initiatives)	7. What external interactions or relationships have impacted the registry's implementation?<br><br>8. What political or health policy factors impacted on the registry's implementation?	i.e. Outside of your hospital, national registry (e.g. IRIS) or NICS-MORU<br><br>All: Does the fact that the registry has been implemented in other countries influence implementation in your hospital/country?<br><br>National lead/development team: Have interactions with outside organisations (ministries of health, professional bodies) influence implementation?<br><br>Nepal: Has involvement in REMAP-CAP influenced the registry or implementation?<br><br>India/Nepal/Malaysia: Did the national IRIS meeting influence the implementation of the registry?<br><br>Does the registry fit with or contradict any health policies? Does the registry fit with or contradict any individual's or group's agenda, political mandate or interests (professional groups, leaders, politicians, institutions)?
The nature of any active implementation process (which incorporates the general principles of effective management in a changing environment)	9. Please describe the challenges (failures) and enablers (successes) to implementation that you've faced. How did you overcome these challenges?<br><br>10. Anything else you would like to say about the implementation of the registry? Where do you see the registry develop in the future?	All: Did the WhatsApp group help? If so, why was it important? Was having examples of how validation works useful?<br><br>Data collectors/Hospital leads: Did you use the user guide? Did the ability to modify the data set, and the layout of the platform help? Was this important?
